# Next generation sequencing misguided the clinical interpretation of the PRSS1 variant in pediatric pancreatitis: a case report

**DOI:** 10.3389/fped.2025.1572366

**Published:** 2025-08-11

**Authors:** Yao Liu, Hongjun Miao, Qin Zhang

**Affiliations:** ^1^ Department of Pharmacy, Children's Hospital of Nanjing Medical University, Nanjing, China; ^2^ Department of Emergency, Children's Hospital of Nanjing Medical University, Nanjing, China

**Keywords:** PRSS1, pediatric, acute pancreatitis, NGS - next generation sequencing, pitfall

## Abstract

Next-generation sequencing (NGS), known as massively parallel sequencing, is transitioning from research tools to a clinical diagnostic methods. Whole-exome sequencing (WES), a specific applications of NGS, has emerged as a valuablefirst-line diagnostic tool for patients with rare diseases and shows promise as aas a comprehensive approach for assessing the prevalence of hereditary pancreatitis. Herein, we present a pediatric case that highlights the pitfalls of false-positive missense variants calls in the PRSS1 gene when using NGS. The patient was ultimately diagnosed with valproic acid-induced acute pancreatitis. Our findings emphasize that relying solely on WES data in epidemiological studies of hereditary pancreatitis might introduce bias, given the difficulties in accurately detecting variants within highly homologous genomic regions.

## Introduction

Variants in the PRSS1 gene, which encodes human cationic trypsinogen have been conclusively associated with autosomal dominant hereditary pancreatitis (1). The p.A16V and p.N29I mutations, both high-penetrance PRSS1 pathogenic variants, may contribute to the multigenic inheritance of a predisposition to pancreatitis. Next-generation sequencing (NGS) enables high-throughput, massively parallel sequencing of thousands to billions of DNA fragments independently and simultaneously. A previous study reported a validation rate of 99.965% for NGS-detected variants, suggesting that routine Sanger sequencing confirmation may not always be necessary (2). Whole-exome sequencing (WES), a prominent application of NGS, is valuable for molecular diagnosis and family genetic counseling (3). It is emerging as a first-line diagnostic tool for patients with rare diseases (4) and holds promise as a comprehensive genetic approach for estimating the prevalence of hereditary pancreatitis (5). We report a case involving false-positive variant calls in PRSS1 identified by NGS in a pediatric patient with pancreatitis. This case underscores the potential for false positives when relying solely on WES for PRSS1 mutation screening.

## Case description

A 6-year-old boy weighing 31 kg was admitted to our Emergency Care Unit after experiencing persistent acute abdominal pain for 23 h. Notably, he had a medical history of viral encephalitis complicated by secondary epilepsy. One day before admission, he underwent a routine follow-up examination at a local hospital and subsequently developed severe abdominal later that evening. An abdominal computed tomography(CT) was performed at the local facility revealed significant enlargement pancreatic enlargement with blurred peripancreatic fat, findings suggestive of acute pancreatitis. As his abdominal pain progressively worsened, he was transferred to our institution for further management. He had no prior history of pancreatitis.

Five months before admission, valproic acid (VPA) was initiated for epilepsy control, with the dosage gradually increased from 300 mg/day–1,000 mg/day. A serum drug concentration measured two months earlier was 75.1 mg/L, which is within the therapeutic range. Concomitant medications included oxcarbazepine and topiramate (both started five months earlier), clonazepam (started two months earlier), lacosamide (started one month earlier), and a low dose of prednisone (1.25 mg/kg/day) for steroid tapering.

On arrival, the patient was afebrile with with stable vital signs, including a heart rate of 112 beats/min and a blood pressure of 135/79 mmHg. Physical examination showed peritoneal irritation, absent bowel sounds, and no skin lesions or palpable abdominal masses. Initial laboratory tests showed leukocytosis (22.31 × 10^9^/L) with neutrophilia (18.45 × 10^9^/L), hyperlactatemia (5.2 mmol/L), and markedly elevated amylase (773 U/L). C-reaction protein (CR*P* < 8 mg/L), procalcitonin (0.084 ng/ml), lipid profile, liver and kidney function, electrolytes, and coagulation parameters were all within normal limits. Urine analysis revealed a significantly elevated urinary amylase level (9,710 U/L).

Following initial fluid resuscitation, the boy was transferred to Pediatric Intensive Care Unit (PICU) for supportive care and management, where he received supportive care including fasting, total parenteral nutrition, empiric antibiotic therapy (ceftriaxone, 50 mg/kg intravenously), analgesia (remifentanil infusion), acid suppression therapy (omeprazole, 40 mg/day intravenously), and anti-pancreatic enzyme therapy (octreotide, 300 μg/day intravenously). On the second hospital day, the patient developed a fever. Concurrently, accompanied by elevated amylase (1,177 U/L) and CRP(143 mg/L). Comprehensive investigations excluded common causes of acute pancreatitis, including biliary obstruction or gallstones (no history or imaging evidence), trauma (denied by the patient and family), infection (initial inflammatory markers were within normal limits, making infectious pancreatitis unlikely), hyperlipidemia, and hypercalcemia (both serum lipid and calcium levels were normal). Liver and kidney function, electrolytes, and coagulation profiles remained unremarkable, effectively excluding systemic diseases as contributing factors. In the absence of other common causes and in light of the recent initiation and dose escalation of VPA, drug-induced pancreatitis was suspected, prompting the discontinuation of VPA therapy. Following its withdrawal, the patient's clinical symptoms and biochemical parameters gradually improved; CRP decreased to 20 mg/L and amylase to 92 U/L within one week. After two weeks, the patient was transferred back to the neurology department without reinitiating VPA treatment.

**Figure 1:**
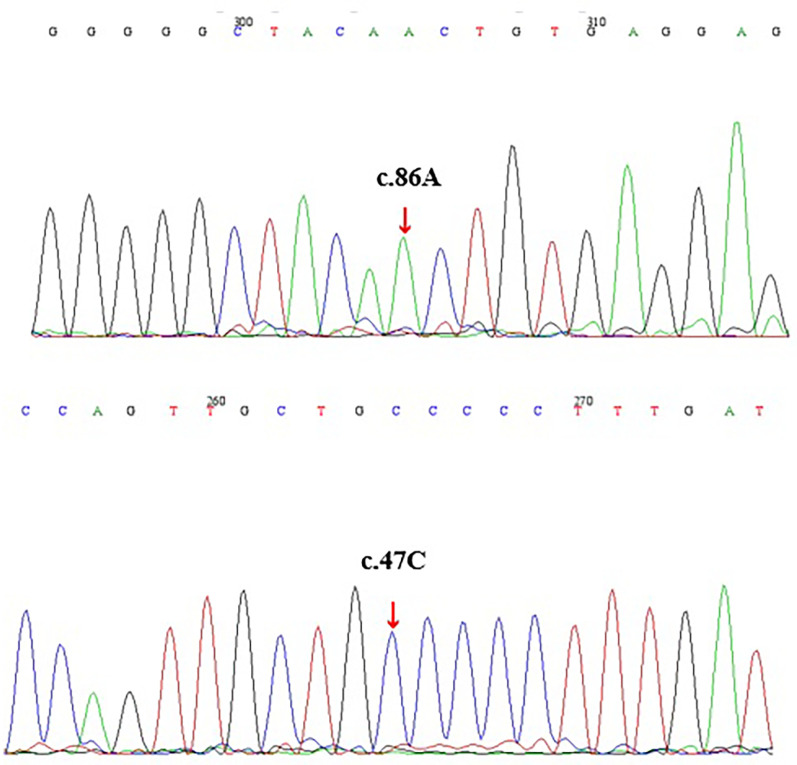
Electropherograms of DNAs from the patient (by sanger sequencing).

Given the patient's family history of pancreatitis (Supplementary Figure S1), hereditary pancreatitis was investigated using WES following informed parental consent and approval from the Ethics Committee of the Children's Hospital of Nanjing Medical University. WES initially identified two heterozygous variants in exon 2 of the PRSS1 gene: c.47C > T (p.A16V) and c.86A > T (p.N29I) (Table 1). However, subsequent validation by Sanger sequencing of all five PRSS1 exons failed to confirm these variants in either the patient or his parents (Figure 1, Supplementary Figures S2, S3). Based on the Naranjo Adverse Drug Reaction Probability Scale (score = 6; Table 2), VPA was determined to be the likely causative agent, leading to a final diagnosis of drug-induced acute pancreatitis. Druing 21 months of follow-up, the child exhibited normal growth and development without recurrence of abdominal pain or other symptoms of pancreatitis.

**Table 1:** Gene variants detected in the patient by whole-exome sequencing.

Gene	Nucleotide Changes	Amino Acid Changes	Clinical Significance^a^	Frequency in gnomAD
CFTR	c.1408G > A	p.V470M	Benign/Likely benign	0.4865
PRSS1	c.508A > G	p.K170E	Benign	0.4163
	c.542G > A	p.S181N	Benign/Likely benign	0.3876
	**c.86A > T**	**p.N29I**	**Pathogenic**	**0** **.** **2831**
	c.617G > C	p.C206S	Benign	0.03190
	c.637G > A	p.V213I	Benign	0.01768
	c.161A > G	p.N54S	Benign	0.02041
	c.40C > G	p.L14V	Uncertain	0.008169
	c.652G > T	p.D218Y	Likely benign	0.006804
	c.146G > T	p.G49V	Benign	0.02693
	c.166C > T	p.Q56X	Uncertain	0.01081
	c.202C > T	p.R68C	Uncertain	0.008504
	c.49C > T	p.P17S	Benign/Likely benign	0.006843
	c.738T > C	p.N246N	Benign	0.5439
	c.486TC	p.D162D	Benign	0.4068
	**c.47C > T**	**p.A16V**	**Conflicting**	**0** **.** **006607**
CTRC	c.356 + 71G > A	-	Benign	0.11168
SPINK1	c.*32C > T	-	Benign/Likely benign	0.07954

^a^ The variant frequency data are obtained from the gnomAD database, while clinical significance annotations are referenced from the ClinVar database.

Bold values indicate variants classified as “Pathogenic” or “Likely Pathogenic” in the ClinVar database.

**Table 2:** Patient's Naranjo adverse reaction probability scale.

Question	Yes	No	Do Not Know	Score
Are there previous conclusive reports of this reaction?	1	0	0	1
Did the adverse event appear after the suspected drug was administered?	2	0	0	2
Did the adverse reaction improve when the drug was discontinued or a specific antagonist was administered?	1	0	0	1
Did the adverse reaction reappear when the drug was readministered	2	−1	0	0
Are there alternative causes (other than the drug) that could on their own have caused the reaction?	−1	2	0	2
Did the reaction reappear when a placebo was given?	−1	1	0	0
Was the drug detected in the blood (or other fluids) in a concentration known to be toxic?	1	0	0	0
Was the reaction more severe when the dose was increased, or less severe when the dose was decreased?	1	0	0	0
Did the patient have a similar reaction to the same or similar drugs in any previous exposure?	1	0	0	0
Was the adverse event confirmed by any objective evidence?	1	0	0	0
Total score				6

## Discussion

The global incidence of acute pancreatitis in the pediatric population is increasing, with an estimated estimated annual incidence of approximately 1 in 10,000 from all causes (6). The etiologies of acute pancreatitis in children are diverse and include infectious diseases, metabolic causes, drugs, and toxins, genetic disorders, obstructive causes, and trauma (6). Among these, genetic factors have received growing attention. Since the PRSS1 gene, which encodes human cationic trypsinogen, was first linked to autosomal dominant hereditary pancreatitis, more than 50 PRSS1 variants have been reported (7). The p.N29I, the second most common PRSS1 mutation, first described in 1997, accounts for nearly 25% of hereditary pancreatitis cases (8). p.A16V, the third most common PRSS1 mutation (9), is primarily associated with idiopathic pancreatitis and shows variable clinical penetrance (10).

**Figure 2:**
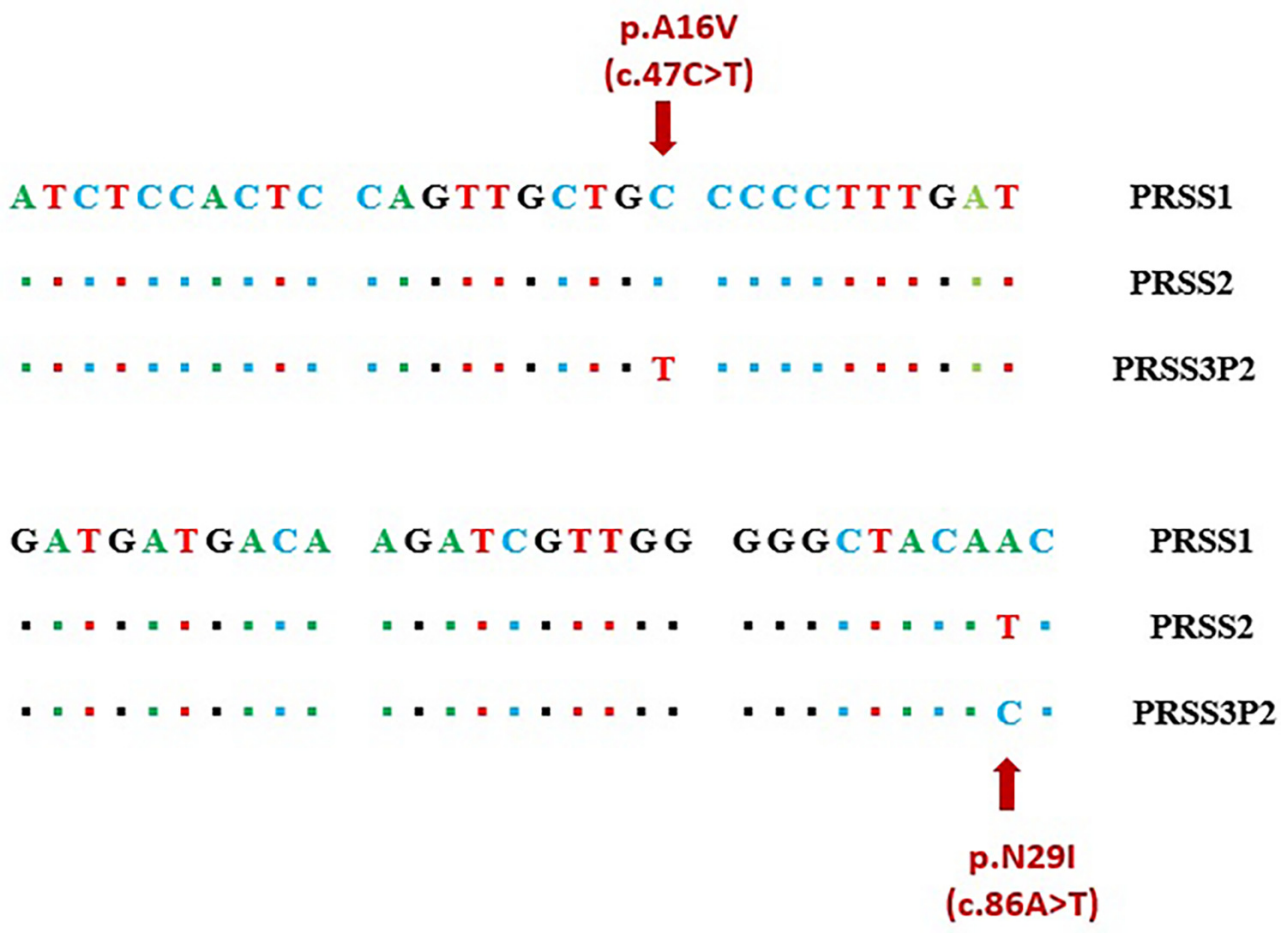
Region of high homology in exon 2 including sequence variants c.47C > T (A16V) and c.86A > T (N29I).

The present study revealed a high rate of false-positive missense variants at the PRSS1 gene when using NGS. These false positives are likely caused by the strong sequence homology among PRSS1, PRSS2, and the pseudogenes PRSS3P3, a known challenge reported in previous studies (11). In particular, the c.86A > T (p.N29I) and c.47C > T (p.A16V) variants are located within regions of about 80 bases pairs that shares very high sequence similarity, making it difficult to accurately align the short reads generated by NGS (Figure 2). This issue leads to the misalignment of sequencing fragments, causing erroneous variant calls (12). Moreover, inherent limitations of NGS technology, such as short read lengths, reliance on reference-based alignment, and intrinsic limitations of variant calling algorithms, further complicate accurate variant detection in these highly homologous regions, frequently resulting in false-positive calls within PRSS1 (13, 14).

To validate the NGS findings, we performed Sanger sequencing of all five PRSS1 exons in the proband and identified only two synonymous variants (c.486T > C/p.D162 = and c.738T > C/p.N246=), while none of the missense variants reported by NGS were detected (Supplementary Figures S2, S3). This clearly demonstrates the false-positive issue associated with NGS in detecting PRSS1 variants and highlights the need for cautious interpretation of NGS results in highly homologous gene regions, supported by confirmatory methods such as Sanger sequencing. Furthermore, we observed that the relatively high allele frequency of p.A16V variant (0.66%) in the gnomAD v2.1.1 database may be influenced by these false-positive calls, potentially explaining the conflicting interpretations of its pathogenicity in the ClinVar database (13).

Drugs account a notable proportion of pediatric acute pancreatitis cases, representing approximately 0.1%–2% (15). In our case, the patient exhibited with severe abdominal pain, markedly elevated serum and urinary amylase levels, and abnormal pancreatic findings on abdominal CT. Based on the Atlanta classification for acute pancreatitis (16), the boy was diagnosed with acute pancreatitis. Comprehensive lipid profile, abdominal CT, and biochemistry excluded other common causes, and long-term use of VPA was identified as the likely etiology. After discontinuing VPA, the patient's symptoms rapidly resolved, and he remained symptom-free over a 21-month follow-up period while continuing treatment with oxcarbazepine, topiramate, clonazepam, and lacosamide, further supporting the diagnosis of drug-induced pancreatitis.

Nonetheless, this study did not assess mutations in the CFTR gene, limiting the ability to exclude other potential genetic factors. Future research should include a comprehensive screening of additional genetic susceptibility genes associated with pancreatitis to better define the genetic background and underlying disease mechanisms.

## Conclusions

The present case highlights the challenges of using NGS to diagnose hereditary pancreatitis. The high sequence homology among genomic regionsposes significant difficulties for accurate genome assembly and variant calling. WES using NGS alone has limitations in reliably detecting mutations in PRSS1. Therefore, confirmation of NGS findings by Sanger sequencing should remain the gold standard for diagnosing hereditary pancreatitis. Furthermore, the use of WES in epidemiological studies of hereditary pancreatitis may introduce data bias, emphasizing the need for cautious interpretation.

## Data Availability

The original contributions presented in the study are included in the article/Supplementary Material, further inquiries can be directed to the corresponding author.
