# Supplementary material for: Next generation sequencing misguided the clinical interpretation of the PRSS1 variant in pediatric pancreatitis: a case report
Source: Front Pediatr. 2025 Aug 11;13:1572366. doi: 10.3389/fped.2025.1572366 (PMC12375464; doi:10.3389/fped.2025.1572366)
Supplement: Supplementary file 1 [file Datasheet1.doc]

**FigureS1. Family Pedigree of the Patient Indicating History of Pancreatitis**


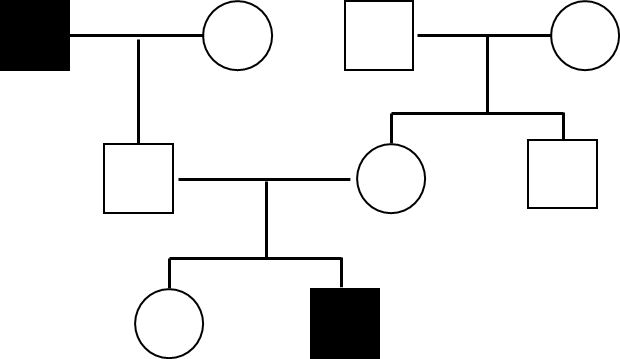


Squares represent males; circles represent females. Filled symbols denote individuals affected by pancreatitis. Only the patient’s grandfather had a history of pancreatitis, while other family members showed no relevant disease history.

**FigureS2. PRSS1 Sanger sequencing.**

**Patient**

1．PRSS1_c.40C


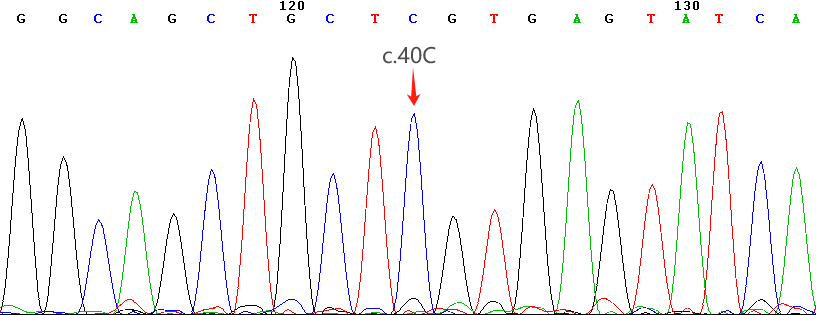


2．PRSS1_c.166C


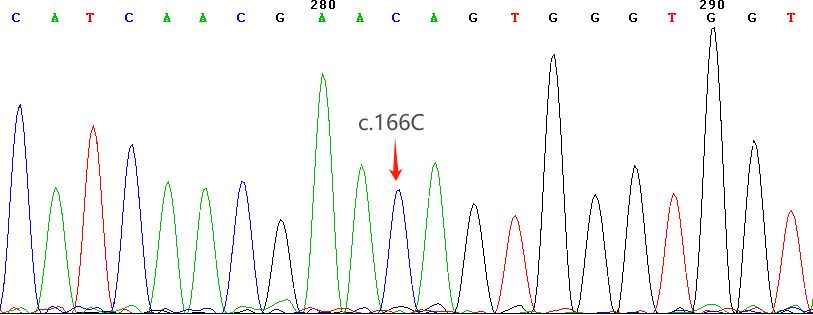


3．PRSS1_202C


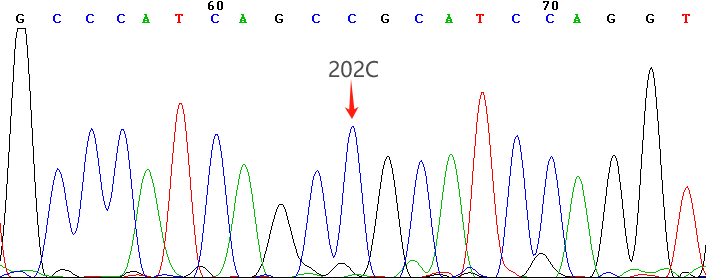


4. PRSS1_c.486T>C (Exon 4)


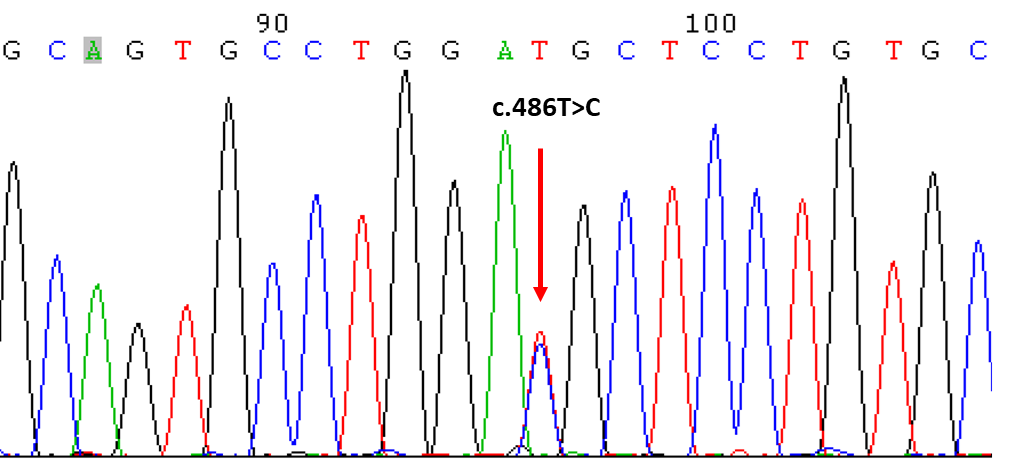


5. PRSS1_c.738 (Exon 5)


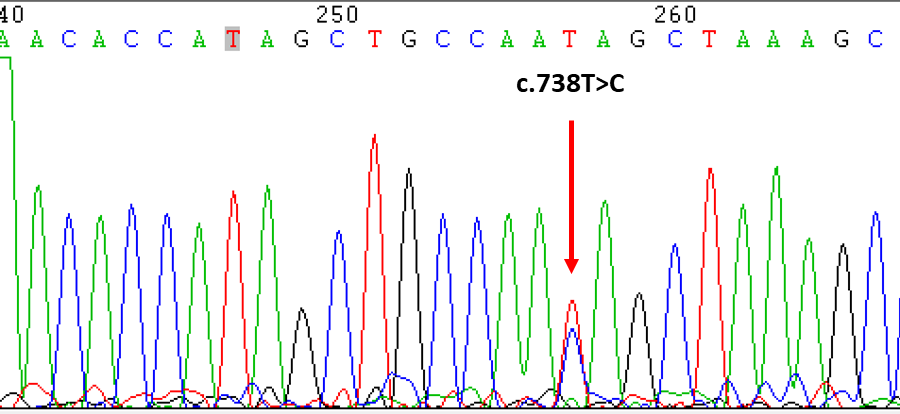


**Patient’s Father**

1、PRSS1_c.40C


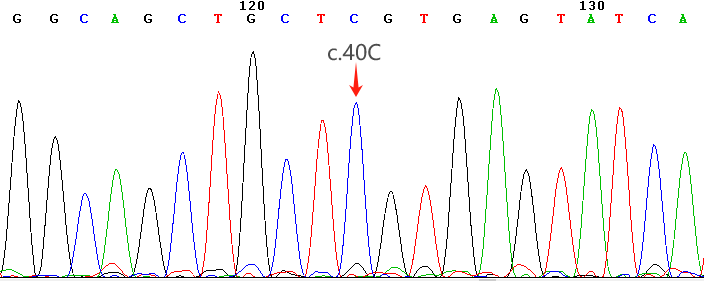


2、PRSS1_c.166C


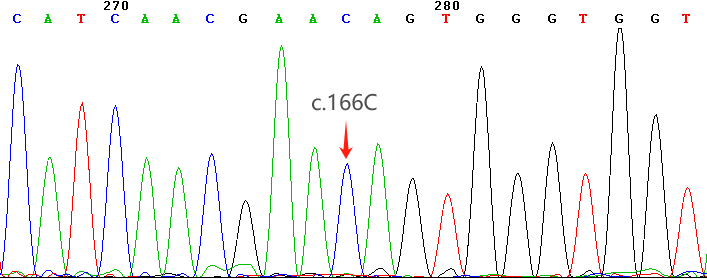


3、PRSS1_202C


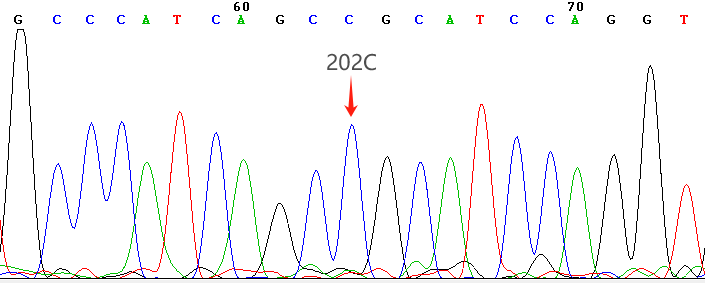


4. PRSS1_c.486T>C (Exon 4)


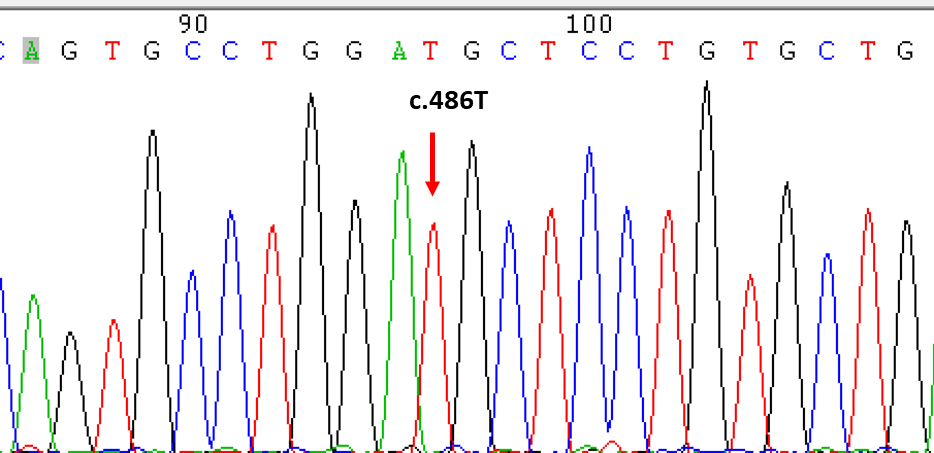


5. PRSS1_c.738 (Exon 5)


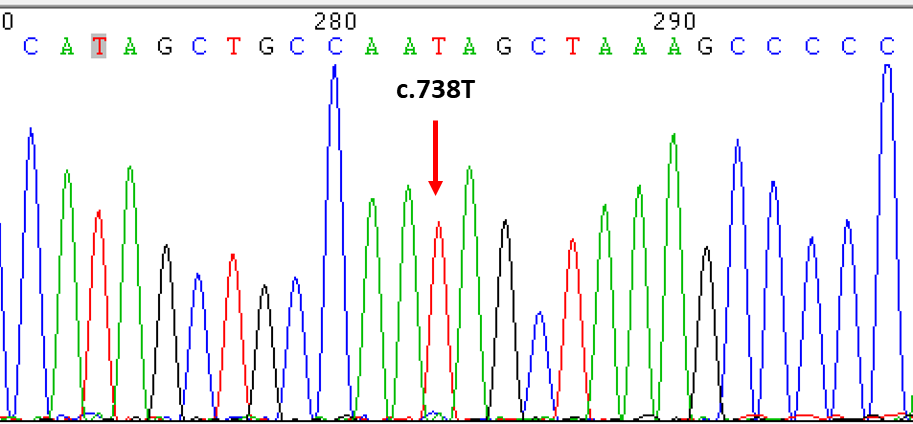


**Patient’s Mother**

1、PRSS1_c.40C


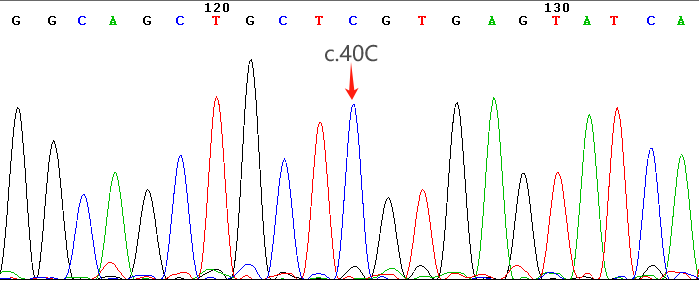


2、PRSS1_c.166C


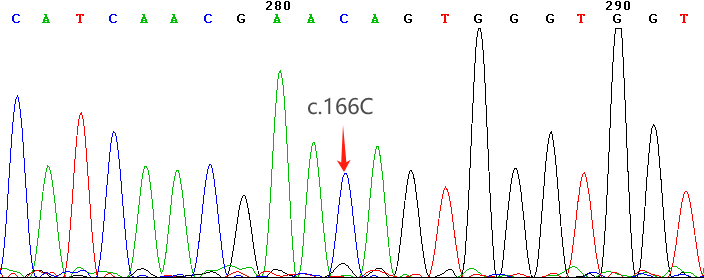


3、PRSS1_202C


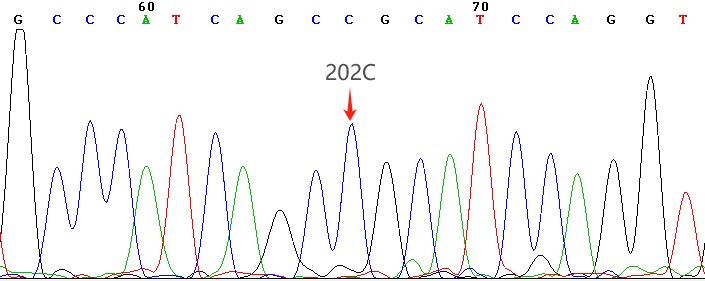


4. PRSS1_c.486T>C (Exon 4)


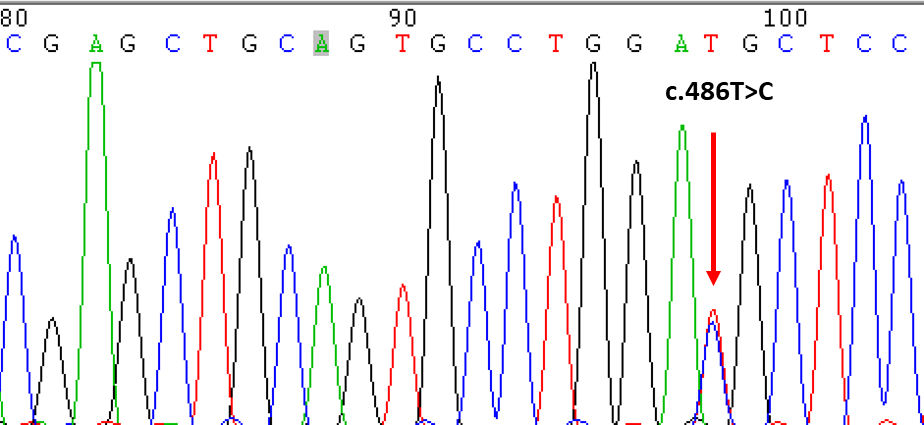


5. PRSS1_c.738 (Exon 5)


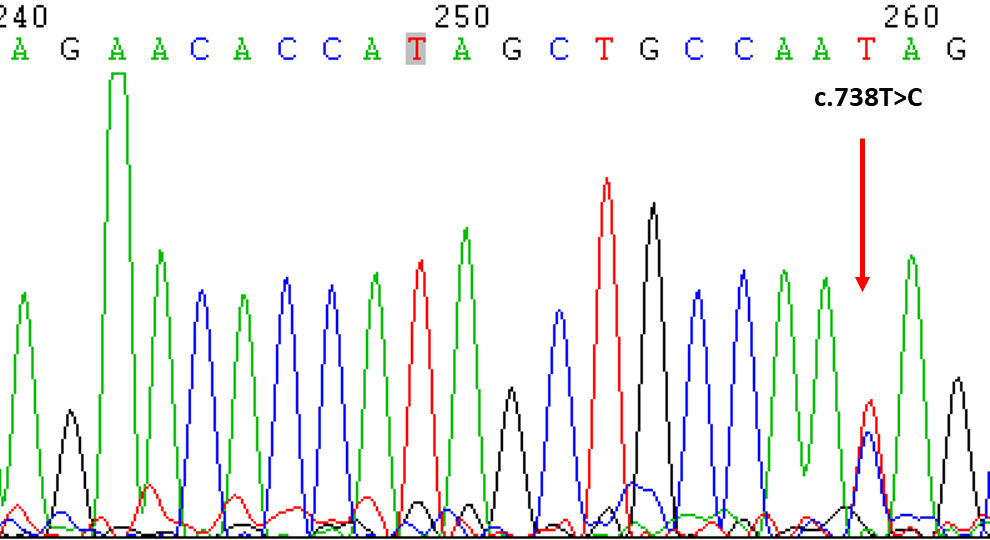


**Patient’s Sister**

1、PRSS1_c.40C


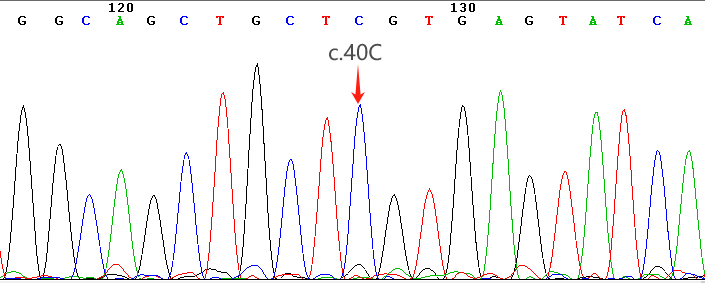


2、PRSS1_c.166C


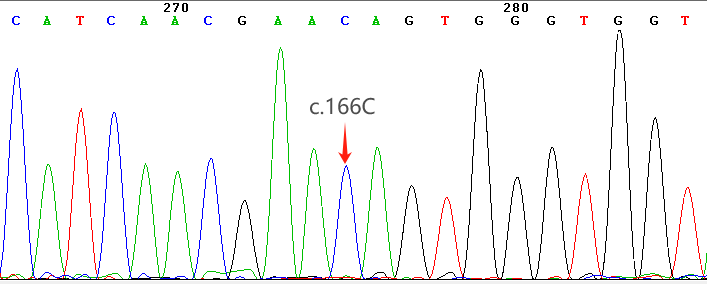


3、PRSS1_202C


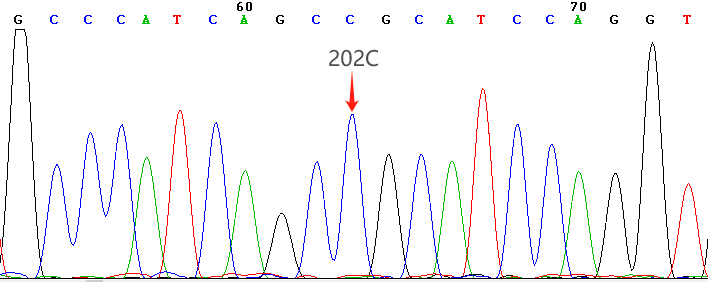


4. PRSS1_c.486T>C (Exon 4)

**
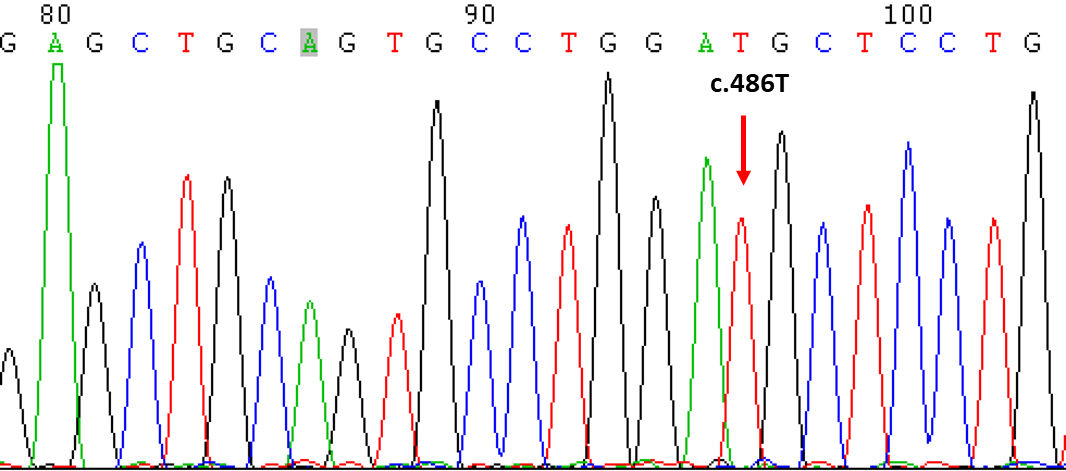
**

5. PRSS1_c.738 (Exon 5)

**
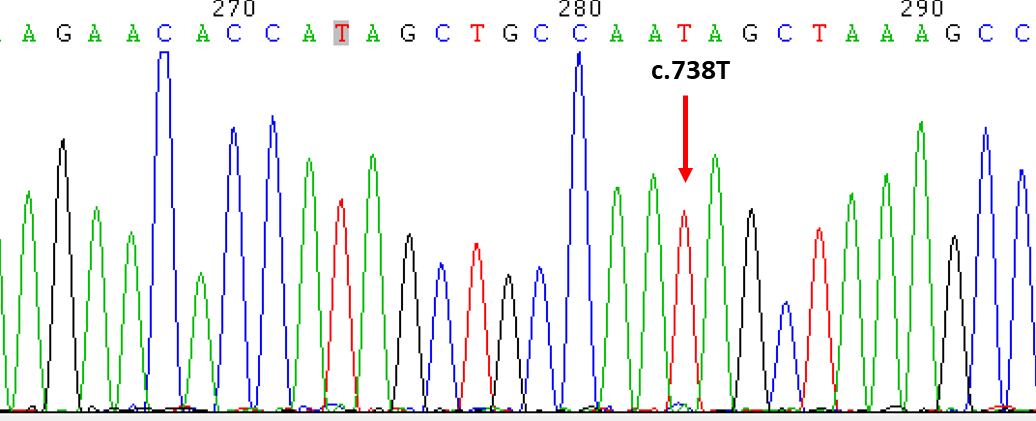
**

**FigureS3. Electropherograms of Sanger sequencing for all 5 exons of PRSS1**

Figure S3-1

**
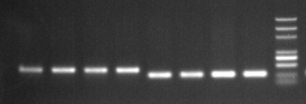
**

From left to right: The Patient-PRSS1_Exon1, The Patient’s Father-PRSS1_Exon1, The Patient’s Mother-PRSS1_Exon1, The Patient’s sister-PRSS1_Exon1, The Patient-PRSS1_Exon2, The Patient’s Father-PRSS1_Exon2, The Patient’s Mother-PRSS1_Exon2, The Patient’s sister-PRSS1_Exon2.

The molecular weight markers (from top to bottom on the right) are: 5000, 3000, 2000, 1000, 750, 500, 250, and 100 bp).

Figure S3-2

**
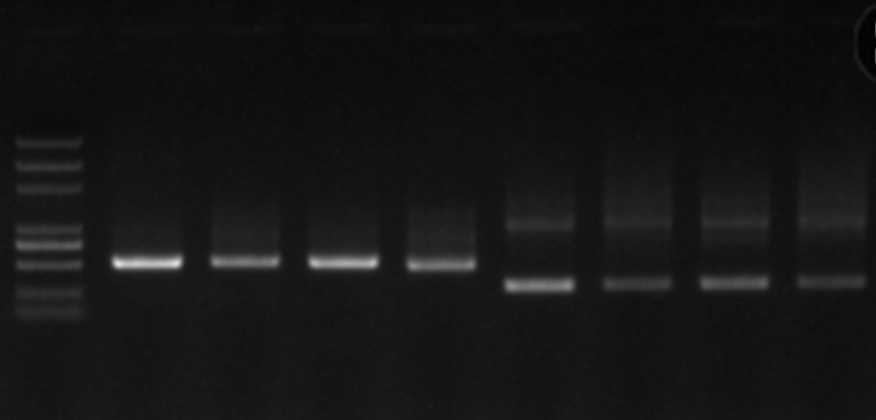
**

From left to right: The Patient-PRSS1_Exon3, The Patient’s Father-PRSS1_Exon3, The Patient’s Mother-PRSS1_Exon3, The Patient’s sister-PRSS1_Exon3, The Patient-PRSS1_Exon4, The Patient’s Father-PRSS1_Exon4, The Patient’s Mother-PRSS1_Exon4, The Patient’s sister-PRSS1_Exon4.

The molecular weight markers (from top to bottom on the left) are: 5000, 3000, 2000, 1000, 750, 500, 250, and 100 bp).

Figure S3-3

**
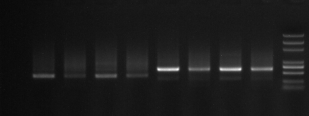
**

From left to right: The Patient-PRSS1_Exon5, The Patient’s Father-PRSS1_Exon5, The Patient’s Mother-PRSS1_Exon5, The Patient’s sister-PRSS1_Exon5, The Patient-CFTR, The Patient’s Father-CFTR, The Patient’s Mother-CFTR, The Patient’s sister-CFTR.

The molecular weight markers (from top to bottom on the right) are: 5000, 3000, 2000, 1000, 750, 500, 250, and 100 bp).
